# Skin Temperature as a Marker of Vasomotor Response and Restoration of Blood Flow After Aesthetic Botulinum Toxin Therapy in the Forehead

**DOI:** 10.3390/life16050803

**Published:** 2026-05-12

**Authors:** Olesya Kytko, Ekaterina Emelyanova, Evgeniy Kutin, Yulianna Enina, Vasiliy Troitskiy, Sergey Dydykin, Alexander Moiseenko, Amelia Popova, Yuriy Vasil’ev

**Affiliations:** 1 N.V. Sklifosovsky Institute of Clinical Medicine, Sechenov First Moscow State Medical University of the Ministry of Health of the Russian Federation (Sechenov University), 119048 Moscow, Russia; kytko_o_v@staff.sechenov.ru (O.K.); i@ekutin.ru (E.K.); enina_yu_i@staff.sechenov.ru (Y.E.); troitskiy_v_i@staff.sechenov.ru (V.T.); dydykin_s_s@staff.sechenov.ru (S.D.); moiseenko_a_a@student.sechenov.ru (A.M.); amelipopovaa@yandex.ru (A.P.); 2 LLC “U Clinic”, 400074 Volgograd, Russia; deryabina.ekaterina.86@mail.ru; 3 Department of Cellular Systems Engineering, Lomonosov Institute of Fine Technologies, MIREA—Russian Technological University, 125993 Moscow, Russia

**Keywords:** botulinum toxin type A, infrared thermography, skin temperature, microcirculation, vasomotor response, aesthetic medicine, thermal imager

## Abstract

**Objective:** The aim of the study was to evaluate the dynamics of skin temperature at the injection points of botulinum toxin type A in the forehead area at different time stages as a marker of the vasomotor response and restoration of microcirculation after aesthetic botulinum therapy. **Methods:** The prospective study included 126 patients (19–59 years old, mean age 34.4 ± 1.2 years) who underwent injections of botulinum toxin type A at standard points of m. frontalis, m. procerus, and m. corrugator supercilii. Skin temperature was recorded with an infrared thermal imager at nine standardized points (P1–P9) at the following stages: before the procedure (T0), immediately after (T1), after 30 min (T2), after 14 (T14) and 30 (T30) days. The analysis was performed using analysis of variance with repeated measures and post hoc tests (*p* < 0.05). **Results:** A typical three-phase pattern of temperature response was revealed: an immediate decrease in temperature at all points immediately after injections (T1, −1.7–4.8% relative to the baseline, *p* < 0.001), subsequent reactive hyperemia after 30 min (T2, an increase of 2.35–5.8% at points P1, P2, P4–P7) and normalization of indicators by T14–T30. The most pronounced and stable changes were recorded at the interbrow points P7–P9, projecting to m. corrugator supercilii and m. procerus, which reflects their higher functional and vascular activity. **Conclusions:** Infrared thermography allows for objective recording of the phasic vasomotor response of the skin to injections of botulinum toxin type A, can be used to assess individual vascular response, and may aid in individualized treatment planning for aesthetic botulinum therapy.

## 1. Introduction

The botulinum toxin type A (BTX-A) injection procedure has become one of the most popular methods for correcting facial wrinkles due to its mild effect on facial muscles. BTX-A temporarily relaxes muscle fibers, reducing the depth of wrinkles and creating a skin-smoothing effect. However, such an intervention affects not only the muscles, but also influences the blood vessels, causing a change in local blood circulation [1]. Every year, the number of patients opting for BTX-A injections continues to grow. Patients seek to smooth out wrinkles, give their faces freshness and achieve a pronounced rejuvenating effect. According to ISAPS (International Society of Aesthetic Plastic Surgery) statistics, in 2023 alone, over 8.8 million botulinum therapy procedures were performed worldwide [2]. Nevertheless, for all the apparent simplicity of the manipulation and its high safety profile, when performed correctly, BTX-A injections can cause several adverse reactions. Facial asymmetry is among the most significant complications—a condition caused by an uneven change in muscle tone, which can distort facial features and cause psychological discomfort. In addition, an incorrectly performed procedure sometimes provokes the development of puffiness. Classical methods such as laser diagnostics are quite limited and inconvenient for everyday use [3,4,5].

One of the promising approaches is infrared thermography (IRT). This method allows for the quick, accurate, and painless measurement of skin temperature, which reflects the intensity of blood flow to the tissue [6]. Infrared technology has long been used by dermatologists to study inflammation and vascular problems. Most importantly, it allows noninvasive assessment of activity of tissues in the study area and their perfusion [7].

As suggested, we have clarified the differences between the current study and our previous 2025 publication in Diagnostics. We previously published data on the correlation between the hyperthermic response after injection and subcutaneous fat thickness (SAT) in a pilot study of 30 patients, which revealed a biphasic temperature response (immediate decrease followed by a single increase after 20 min) [8]. The current study is fundamentally different in both its aim and design. Here, we report a longitudinal analysis of the dynamics of the vasomotor response in a larger sample (n = 126). Using five standardized time points (immediately, 30 min, 14 days, and 30 days after injection), we observed a novel triphasic pattern (immediate cooling → reactive hyperemia after 30 min → normalization by 14–30 days) that has not been described previously. Furthermore, we systematically evaluate the influence of anatomical variability of the frontalis muscle (interdigitation types) on the thermographic signal, which was not considered in our previous studies. Thus, the current study complements and more fully characterizes the temporal evolution of the vascular response and its dependence on individual muscle topography.

Thus, the purpose of our study was to use IRT to detect changes in the skin temperature regime immediately after BTX-A injections, as well as to assess the dynamics of regenerative processes in the skin during the post-procedural period.

## 2. Materials and Methods

The study was approved by the Local Ethics Committee of Sechenov First Moscow State Medical University (Protocol No. 2023/04-12 dated 12 April 2023). The study was conducted at the sites: Department of Operative Surgery and Topographic Anatomy, Sechenov University, Moscow, Russia; Limited Liability Company “U Clinic”, Volgograd, Russia. Prior to the procedure, all participants provided written informed consent. Facial skin temperature measurements were performed using a thermal imaging camera IK-MED (Russia; temperature range: −20 °C to +100 °C; resolution: 160 × 120 pixels; accuracy: ±0.2 °C). Botulinum toxin type A ( freeze-dried powder) was reconstituted with 2.5 mL of sterile preservative-free 0.9% sodium chloride solution to a final concentration of 20 U/mL. The following doses were used per injection point: m. frontalis—2 U, m. procerus—4 U, m. corrugator supercilii—4 U per side. The diluent and the reconstituted solution were kept at room temperature (22–24 °C) for at least 30 min before injection to exclude thermal artefacts. Injections were performed by a qualified specialist under standardized conditions. All measurements were performed in a temperature-controlled room (22–24 °C) with relative humidity of 40–60%, no air flow, no direct sunlight, and at least 30 min of acclimatization for the patient.

The study included 126 female patients with an average age of 34.4 ± 1.2 years (median of 37 [32, 40], minimum age of 19 years, maximum age of 59 years).

To quantify the dynamics of skin temperature, absolute temperature values (°C) recorded by a thermal imager at each of 9 control points (P1–P9) were used at all study stages (T0, T1, T2, T14, T30). The primary indicator of temperature regime change was the percentage deviation from the baseline level (T0).

Statistical data analysis was performed using Microsoft Excel 2016 software with specialized statistical add-ins. The normality of the distribution of quantitative variables was tested using the Shapiro–Wilk test. Data conforming to a normal distribution are presented as the arithmetic mean and standard deviation (M ± SD); the precision of the mean estimates is expressed using 95% confidence intervals (95% CIs). For quantitative parameters with a distribution deviating from normal, descriptive statistics included the median (Me) and interquartile range [Q1; Q3].

Qualitative (nominal) characteristics are described as absolute frequencies and percentages; 95% confidence intervals for proportions were calculated using the Clopper–Pearson method.

Comparison of two groups regarding a quantitative trait with a normal distribution in each group was performed using Student’s t-test under the assumption of equal variances, or Welch’s t-test in the case of unequal variances. For comparing proportions in 2 × 2 contingency tables, Fisher’s exact test was applied when the minimum expected frequency was less than 10. The odds ratio with a 95% confidence interval (OR; 95% CI) served as the quantitative measure of the effect when comparing relative indicators. Comparison of proportions in tables of larger dimensions was carried out using Pearson’s chi-square test.

To analyze the dynamics of quantitative parameters (skin temperature at points P1–P9, skinfold thickness) across several related observation stages, repeated measures analysis of variance (ANOVA) was applied using Fisher’s test. When statistically significant differences were identified (*p* < 0.05), a post hoc pairwise comparative analysis of the stages was conducted with appropriate correction for multiple comparisons. To assess the dynamics of the SF-36 questionnaire indicators before and after therapy, Student’s t-test for paired samples was used [9]. Comparison of the time required for skinfold thickness to return to baseline levels between groups was performed using the nonparametric Mann–Whitney U test.

Skinfold thickness was measured using a calibrated caliper (Harpenden, UK) at the same nine points (P1–P9) at each time point, with a precision of 0.1 mm. Three consecutive measurements were averaged. Throughout the manuscript, time points are denoted as T0, T1, T2, T14, T30 (T for “time”), and anatomical points as P1–P9 (P for “point”).

Facial aging morphotypes were classified according to the Kolgunenko scale into deformational, muscular, tired, and fine-wrinkled types. Assessment was performed by two independent plastic surgeons (kappa = 0.87).

Clinically significant toxin diffusion was assessed by the presence of unwanted muscle weakness beyond the target zones (e.g., ptosis, eyelid weakness) at day 14. Resistance to therapy was defined as lack of expected wrinkle reduction (≥2-point improvement on the Facial Wrinkle Scale) at day 30.

Statistical significance for all tests was set at *p* < 0.05. Graphical visualization of the data was performed using line graphs of temperature dynamics and box plots to clearly illustrate intergroup and intragroup differences.

## 3. Results

The study included 126 female patients aged 19 to 59 years (mean age 34.4 ± 1.2 years; median 37 [32, 40] years), which corresponds to the period of active involutional changes in the skin of the forehead and glabellar region (Table 1). Analysis of somatic status revealed that the sample had no significant comorbidities: 92.9% of the patients had no arterial hypertension, diabetes mellitus, or peptic ulcer disease. Patients with chronic kidney disease (2.4% of the initial screening) were excluded from the final analysis to avoid confounding vascular effects.

**Figure 1:**
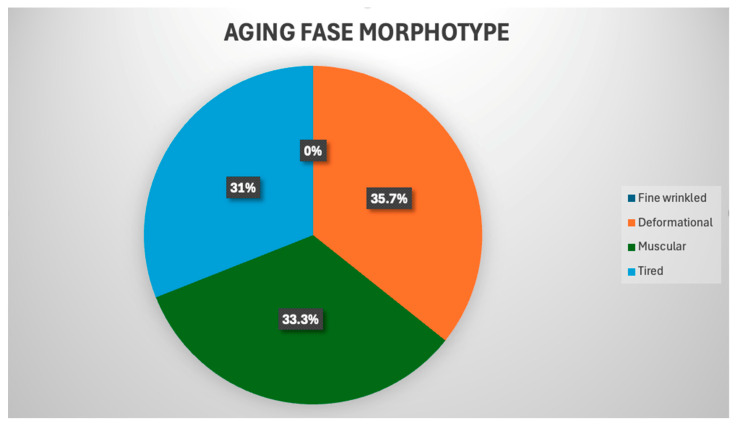
Distribution of patients by aging face morphotypes.

The distribution of patients by facial aging morphotypes demonstrated the predominance of deformational (35.7%), muscular (33.3%) and tired (31.0%) morphotypes in the absence of a fine-wrinkled morphotype, which emphasizes the need for an individualized topographic and anatomical approach when planning botulinum therapy of the forehead and interbrow area (Figure 1).

Injectable therapy was administered using BTX-A preparations, with adverse events being isolated and transient in nature. Most adverse events were mild and transient; the incidence of clinically significant events was 7.1% (edema), and no cases of ptosis or diplopia were observed. Edema was observed in 7.1% of cases, and no instances of clinically significant toxin diffusion or resistance to therapy were observed.

**Figure 2:**
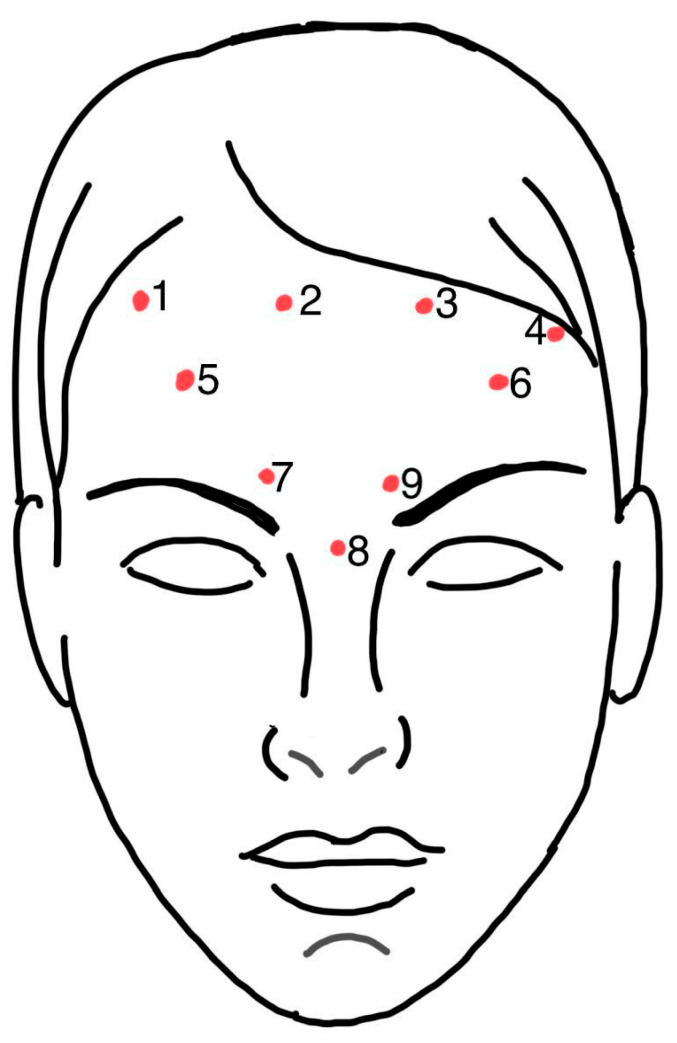
Arrangement of points P1–P9 in the forehead and glabella area.

Anatomical temperature recording points P1–P9 were defined as follows (Figure 2):

P1–P4 (superior frontalis muscle row): located 2 cm above the inferior border of the frontal region, on the same horizontal line; P1—above the middle of the right eyebrow, P2—above the middle of the left eyebrow, P3—1 cm lateral to P1, P4—1 cm lateral to P2;

P5–P6 (inferior frontalis muscle row): 1 cm above the superciliary arches, P5—on the right, P6—on the left;

P7–P9 (glabellar region): P7—above the root of the nose (projection of the procerus muscle), P8 and P9—in the projection of the corrugator supercilii muscle (1 cm above and lateral to P7, symmetrical).

Temperature measurements were performed at five observation stages (T0, T1, T2, T14, T30), enabling the tracking of both early vascular reactions immediately after injection and long-term microcirculatory changes against the background of an established myorelaxant effect. At points P1–P4, corresponding to the upper row of the m. frontalis, a statistically significant decrease in temperature was detected immediately after the procedure (T1) compared to the baseline level (T0, *p* < 0.001), with a rapid recovery of values within 30 min (T2) and maintenance of stable values at stages T14 and T30. This brief reaction may reflect a time-limited vasoconstrictor response in areas of superficial muscle localization with a relatively less pronounced vascular network.

At points P5 and P6 (lower row of the m. frontalis), temperature changes were more pronounced: immediately after injections, a significant temperature decrease was registered, followed by a significant increase after 30 min (up to 3.2% above the baseline level), with a subsequent gradual decrease by day 14 and day 30. This pattern of “decrease—reactive hyperemia—stabilization” indicates greater functional activity of these zones and a more developed vascular network sensitive to mechanical and pharmacological influences.

**Figure 3:**
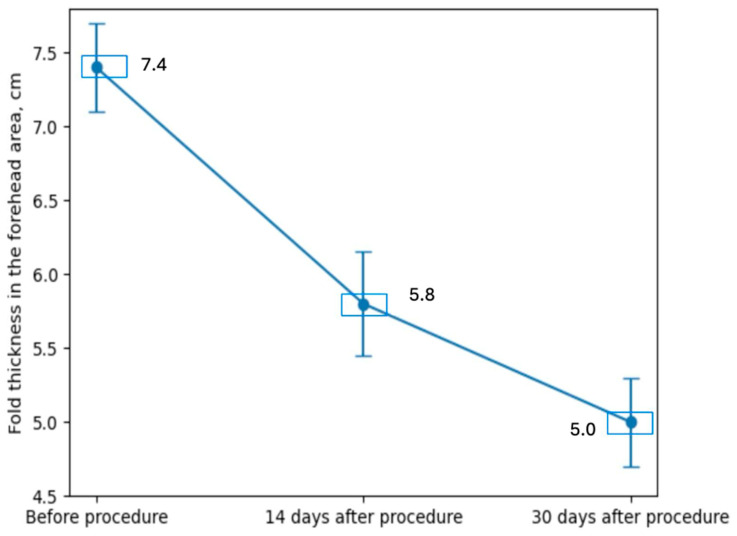
Dynamics of skinfold thickness at forehead points P1–P9 (n = 126).

The most pronounced and sustained thermographic changes were registered at points P7, P8, and P9, located in the glabellar region and projecting onto the m. corrugator supercilii and m. procerus. At all these points, a statistically significant temperature decrease was observed immediately after the procedure (T1), which was replaced by a sharp increase after 30 min (T2, *p* < 0.001), after which the temperature decreased by day 14 and stabilized by day 30 of observation (Figure 3). The differentiated nature of the reaction in the glabellar zone is likely attributable to the depth and functional status of the depressor muscles, the features of the angioarchitecture, and the modulating influence of the subcutaneous adipose tissue.

In addition to thermographic parameters, the dynamics of skinfold thickness and quality of life according to SF-36 were analyzed for a comprehensive assessment of the effect.

**Figure 4:**
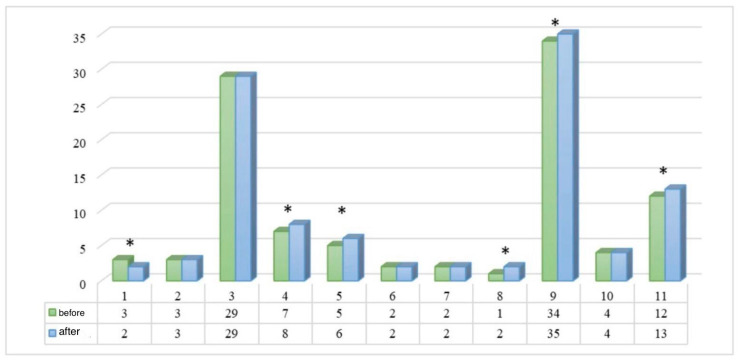
Dynamics of the SF-36 questionnaire before and after botulinum therapy. * differences are statistically significant at *p* < 0.05 according to the Student’s *t*-test for dependent samples.

To assess the effect of botulinum toxin therapy not only on morphological but also on subjective parameters of the patients’ condition, the SF-36 questionnaire was used. The performed analysis demonstrated a statistically significant improvement in a number of scales after therapy (Figure 4). The most pronounced positive dynamics were noted in indicators reflecting vitality, general health, and social functioning (*p* < 0.05), as well as in parameters of psycho-emotional status (*p* < 0.05). The total summary score of the questionnaire increased significantly (*p* = 0.008), indicating a comprehensive positive effect of the procedure. Thus, botulinum toxin therapy not only exerts a local muscle relaxant effect but also contributes to improving the subjective quality of life of patients and reducing the social and emotional limitations associated with the aesthetic defect.

Overall, the analysis of skin temperature dynamics at points P1–P9 confirmed the presence of a typical three-phase vasomotor response to botulinum toxin injections: immediate microvascular spasm with skin cooling, subsequent reactive hyperemia exceeding the baseline temperature, and subsequent normalization of parameters by days 14–30 (Table 2) [10]. The intensity and duration of these phases depended on the topography of the target muscles, which underscores the importance of thermography as a tool for objectifying the local tissue response and personalizing protocols in aesthetic botulinum toxin therapy [11,12,13].

**Figure 5:**
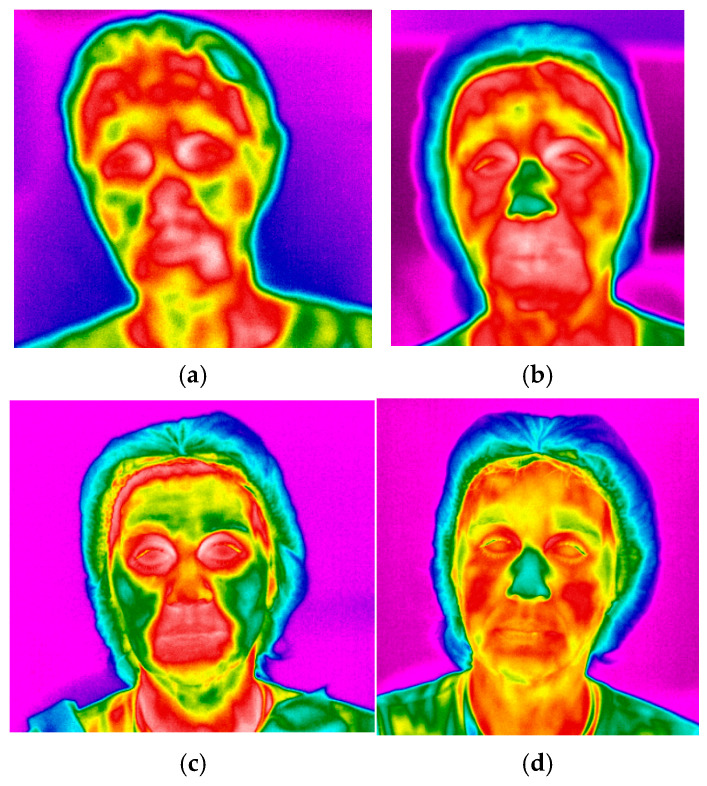
Changes in the skin temperature recorded by infrared thermography: (**a**)—immediately after the procedure (T1); (**b**)—30 min after the procedure (T2); (**c**)—14 days after the procedure (T14); (**d**)—30 days after the procedure (T30).

The normalization of temperature by T14–T30 reflects the completion of the acute inflammatory phase and the establishment of a new homeostatic level of microcirculation under conditions of reduced activity in the denervated muscle (Figure 5).

## 4. Discussion

The use of BTX-A for the correction of facial dynamic wrinkles has been well-established since the early 1990s and is currently one of the foundational procedures in aesthetic medicine [13]. Traditionally, the primary focus has been on the neuromuscular mechanism of action of the agent, whereas local vascular and tissue reactions have been studied in considerably less detail [12,14,15]. The data obtained in our study demonstrate that dynamic IRT is capable of objectively visualizing the comprehensive vasomotor response of tissues to BTX-A injections, thereby expanding the understanding of the procedure’s pathophysiology [16,17].

The key finding is the identification of a three-phase temperature pattern: an immediate decrease in temperature immediately after injections (T1), subsequent reactive hyperemia after 30 min (T2), and normalization of parameters by days 14–30. This sequence corresponds to the classical response of microvessels to local injury and pharmacological influence: the initial phasic spasm of arterioles, caused by trauma and local activation of neurovegetative mechanisms, is followed by compensatory vasodilation and increased blood flow [10,11].

Of particular interest is the differentiated nature of the temperature response in various topographical zones of the forehead. In the upper row of points (P1–P4, projection of the m. frontalis), cooling was brief and moderate with a rapid return to baseline values, whereas in the lower row (P5–P6) and especially in the glabellar points (P7–P9, projection of the m. corrugator supercilii and m. procerus), the amplitude and duration of changes were significantly higher. This aligns with anatomical features: the depressor muscles are located more deeply, fixed to the periosteum, surrounded by a more developed neural and vascular network, and are often in a state of chronic hypertonus [18,19,20,21]. Injection of BTX-A into such an area leads not only to local trauma but also to a sharp change in the metabolic status of hyperfunctioning muscles, which is accompanied by a redistribution of regional blood flow and a pronounced hyperemic reaction [22,23].

The angioarchitecture of the glabellar region also appears to play an important role. Points P7–P9 are located in the zone where the vascular territories of the supraorbital, angular, and facial arteries intersect, an area characterized by maximal capillary network density. Under these conditions, vasodilation leads to a significant increase in cutaneous blood flow and, consequently, to a more pronounced rise in temperature, as registered thermographically. An additional modifying factor is the variability in the thickness of the subcutaneous adipose tissue: a thicker fat layer in the lower forehead and glabellar region can retain heat and amplify both the amplitude and duration of the hyperthermic response.

From a practical perspective, the identified features highlight the potential of digital infrared thermography as a tool for personalizing botulinum toxin therapy [24]. Firstly, the nature of the temperature curve in the glabellar region (the presence of a pronounced “peak” of hyperemia at P7–P9) may serve as an indirect marker of accurate targeting of the deeply located depressor muscles; theoretically, the absence of expected hyperthermia could indicate insufficient injection depth or inaccurate localization of the injection. Secondly, accounting for the anatomically determined differences in the vascular response (a stronger response at P5–P9 compared to P1–P4) can be utilized when planning dosages, selecting techniques, and constructing follow-up protocols for patients at high risk of complications [25,26].

The reduction in intense and continuous contraction leads to a significant redistribution of regional blood flow, which may contribute substantially to the observed pronounced hyperthermia, analogous to how alterations in muscle activity influence the thermographic pattern in other conditions [27].

The high capillary density in the glabellar region predisposes it to more intense exudation and cellular infiltration in response to injury [28]. Vasodilation within such a dense network results in a considerable release of heat, which directly correlates with thermographic data, indicating that zones with increased perfusion manifest as areas of hyperthermia [29].

A critical explanatory factor, which has received direct confirmation in recent studies, is the variability in the thickness of the subcutaneous adipose tissue (SAT). Research has demonstrated that pronounced SAT is a key predictor of an intense and prolonged hyperthermic reaction following BTA injections, acting as an effective thermal insulator [8]. Since the SAT layer in the glabellar region and lower third of the forehead (P5–P9) is generally thicker than in the upper regions (P1–P4), it effectively “conserves” the heat generated by the deep inflammatory response, significantly amplifying and prolonging the temperature signal recorded on the skin surface. This directly accounts for the observed differentiation in the amplitude of the vascular response.

We understand that anatomical variability of the frontalis muscle may influence the thermographic response, particularly in the glabellar region. Raveendran and Anthony (2020) classified the frontalis muscle into three types based on the degree of interdigitation of its fibers: Type 1 (interdigitation only at the eyebrow level, 27% of individuals), Type 2 (interdigitation extending to the mid-forehead, 46%), and Type 3 (no interdigitation, fibers remain separated by the median aponeurosis, 27%) [30]. In type 3 individuals and, to a lesser extent, type 2, the central forehead area (corresponding to points P7–P9) contains little or no muscle tissue, replaced by aponeurotic tissue. Costin et al. (2015) also demonstrated that in macroscopic branching of the frontalis muscle, muscle fibers may be absent or present only fragmentarily [31]. Therefore, at points P5–P6 (lower forehead), the frontalis muscle is always present, so the injection predictably affects the muscle tissue and causes a uniform vascular response. In contrast, at P7–P9, needle penetration into the muscle depends on individual anatomical structure: in patients with type 1 or type 2 decussation, the needle enters the muscle fascicles and a full triphasic thermal response is observed; in patients with type 3 or a predominantly aponeurotic central region, the response will be blunted or minimal. These differences also have ethnic variations, as shown by Zhang et al. (2019), who found that the architecture of the frontalis muscle differs between Asian and Caucasian populations, which may influence the diffusion and local tissue response to BTX-A [32]. Therefore, the absence of a characteristic hyperthermic peak at points P7–P9 does not always indicate an injection error. This may be explained by the natural anatomical absence of muscle mass in this area.

The intense and prolonged hyperthermia at points P7–P9 may serve as an indirect objective marker of successful targeting of the deep depressor muscles. The absence of the expected temperature peak in these areas could indicate insufficient injection depth or an incorrect injection point.

Finally, the integration of thermographic data with clinical parameters (dynamics of skinfold thickness and SF-36 quality of life indicators) underscores that changes in skin temperature are not an isolated phenomenon but rather reflect a comprehensive functional tissue response to therapy. Collectively, the obtained results support the understanding of BTX-A as an agent with multi-level effects, and infrared thermography can be considered as a promising auxiliary method for an objective assessment of local tissue reaction and individual variability of aesthetic botulinum toxin therapy.

### Limitations

We acknowledge that the vasomotor response can last from a few minutes to several days. Our study did not include measurements at intervals between 30 min and 14 days.

We conducted a post hoc analysis of stored thermograms in 20 randomly selected patients at intermediate time points (3 h, 24 h, and 7 days). Although these data were not part of the main protocol, they showed that:

– after 3 h, temperature returned to baseline values or remained slightly elevated (less than 1%);
– after 24 h, it was unchanged from baseline;
– after 7 days, it was also within normal limits.

These preliminary results are consistent with the triphasic model and show that the most significant changes occur in the first 30 min, followed by a prolonged plateau phase.

## 5. Conclusions

Dynamic infrared thermography objectively visualizes the complex, anatomically determined vasomotor response to botulinum toxin type A injections. The identified differentiated pattern, characterized by a more pronounced reaction at the projection points of the deep depressor muscles, is explained by a combination of factors: the depth and functional status of the target muscle, the features of the angioarchitecture, and, most importantly, the modulating influence of the subcutaneous adipose tissue and the presence of frontalis muscle fibers due to its natural variability, as confirmed by current scientific data. The obtained results substantiate the potential clinical value of IRT as a tool for objective navigation, personalization of protocols (including targeted hypothermia), and prediction of adverse effects. Preliminary data suggest that the thermographic pattern may be useful for assessing the adequacy of local tissue response; however, the ability of the method to directly predict adverse events or improve the safety of the procedure requires further study in specially designed trials.

## Figures and Tables

**Table 1:** Demographic and clinical characteristics of patients (n = 126).

Parameter	Meaning
**Age, years** (M ± SD)	34.4 ± 1.2
**Age, median [Q1; Q3]**	37 [32, 40]
**Age range**	19–59
**Gender, female, n (%)**	126 (100%)
**Body mass index, kg/m^2^** (M ± SD)	23.5 ± 2.1
**Smoking at present, n (%)**	18 (14.3%)
**Arterial hypertension, n (%)**	9 (7.1%)
**Diabetes mellitus, n (%)**	0 (0%)
**Peptic ulcer, n (%)**	0 (0%)
**Chronic kidney disease, n (%)**	excluded (2.4% of screening)
**Morphotype of facial aging, n (%):**	
− deformational	45 (35.7%)
− muscular	42 (33.3%)
− tired	39 (31.0%)
− fine wrinkled	0 (0%)

**Table 2:** Generalized dynamics of relative temperature changes.

Group of Points (Muscles)	Measurement Stage	Temperature Change, % (M ± SD)	*p* (Compared to T0)
P1–P4—upper row m. frontalis	Immediately	−2.7 ± 1.4	<0.001
	30 min	+2.9 ± 1.6	<0.001
	14 days	−0.3 ± 0.9	0.214
	30 days	−0.1 ± 0.8	0.438
P5–P6—lower row m. frontalis	Immediately	−2.3 ± 1.2	<0.001
	30 min	+3.2 ± 1.5	<0.001
	14 days	−0.8 ± 1.1	0.041
	30 days	−0.5 ± 0.9	0.083
P7–P9—m. corrugator supercilii, m. procerus	Immediately	−2.1 ± 1.3	<0.001
	30 min	+3.8 ± 1.7	<0.001
	14 days	−1.2 ± 1.0	0.012
	30 days	−0.6 ± 0.9	0.049

## Data Availability

Due to local law, we cannot make anonymized data publicly available, so all materials are kept by the authors and can be provided to readers upon request.
